# Scrub Typhus in Northern India: An Overview of Epidemiology, Risk Factors, Seasonal Patterns, and Public Health Implications

**DOI:** 10.7759/cureus.106498

**Published:** 2026-04-05

**Authors:** Arjun S Bisht, Anita Pandey, Peetam Singh

**Affiliations:** 1 Microbiology, Subharti Medical College, Swami Vivekanand Subharti University, Meerut, IND

**Keywords:** acute febrile illness, endemic, north india, orientia tsutsugamushi, scrub typhus

## Abstract

Scrub typhus, caused by *Orientia tsutsugamushi* and transmitted by larval trombiculid mites, has become a major re-emerging infectious disease across Northern India, especially in the sub-Himalayan region. This review highlights current evidence on its epidemiology, regional variations, risk factors, and seasonal trends using surveillance, hospital-based, and outbreak data. The North Indian states of Himachal Pradesh and Uttarakhand, along with adjoining regions of Uttar Pradesh, report many cases in hospital settings and outbreaks in the community, with a seasonal peak from July to November coinciding with climatic conditions that likely favour vector proliferation. The transmission of scrub typhus is closely associated with occupational exposure in agriculture and forestry, proximity to scrub vegetation and rodents, and behavioural practices that increase contact with mites. The clinical presentation often overlaps with other febrile illnesses, complicating the diagnosis, and due to limited diagnostic infrastructure, scrub typhus is likely underreported, particularly in districts with low diagnostic capacity, resulting in delayed treatment. The public health implications include recurrent monsoon-related outbreaks, a heavy healthcare burden, risks of antimicrobial misuse, and antimicrobial stewardship concerns, especially when the diagnosis is uncertain. This review highlights the need for integrated control strategies, such as district-level diagnostics and surveillance integration, along with personal protection, environmental management, improved diagnostics, community awareness, sustained surveillance, and research on vector ecology, strain diversity, and vaccine development. Strengthening multi-sectoral collaboration holds the greatest potential to reduce the growing burden and mortality from scrub typhus in Northern India.

## Introduction and background

Scrub typhus is an acute undifferentiated febrile illness (AUFI) caused by the obligate intracellular bacterium *Orientia tsutsugamushi* and transmitted through the bite of infected larval forms of trombiculid mites, known as chiggers. AUFI is a medical condition characterised by the sudden onset of fever (≥38°C or ≥100.4°F) lasting for less than 2 weeks and not attributed to a specific cause after thorough clinical evaluation and laboratory testing. Scrub typhus is a neglected tropical infection causing substantial morbidity and mortality, particularly across the Asia-Pacific region, including India. Scrub typhus has become a major public health concern in recent decades, especially in the sub-Himalayan region, with increasing detection and outbreaks being reported, probably due to favourable environmental conditions. Its epidemiology is complex, with significant regional variation shaped by ecological, climatic, behavioural, and socioeconomic factors. Understanding its distribution, risk factors, and seasonal patterns is essential for implementing targeted prevention strategies and reducing disease burden.

Historically, scrub typhus was first recognized during World War II in Northeast India among troops exposed to scrub vegetation. Since then, growing evidence from surveillance and hospital-based studies has revealed its wider geographic distribution across Himachal Pradesh, Uttarakhand, Jammu and Kashmir, Punjab, and nearby states [1-4]. This narrative review synthesises the epidemiology and regional variations of scrub typhus in Northern India, discusses transmission risk factors, outlines seasonal patterns, and explores the public health challenges and implications of controlling this re-emerging infectious disease.

## Review

Methodology

The relevant literature was searched in PubMed and Google Scholar for scrub typhus in North India using the keywords scrub typhus AND North India, *Orientia tsutsugamushi* AND North India, scrub typhus AND Uttar Pradesh, scrub typhus AND Punjab, scrub typhus AND Himachal Pradesh, scrub typhus AND Uttarakhand, scrub typhus AND Haryana, and scrub typhus AND Jammu and Kashmir. The literature search covered a period of 15 years, from 2010 to 2025. Meta-analyses, systematic reviews, and duplicate articles were excluded from the study.

Epidemiology and regional distribution in Northern India

Northern India includes diverse ecological zones such as the Himalayan foothills, river valleys, plains, and urban areas, each exhibiting distinct scrub typhus epidemiology. The sub-Himalayan belt is a well-documented hotspot, with Himachal Pradesh reporting some of the highest scrub typhus positivity rates in the country, reaching up to 96% among febrile illness cohorts, as highlighted in a systematic review by Devasagayam E et al. [1,5,6]. Uttarakhand and neighbouring Uttar Pradesh also bear a significant burden of scrub typhus, with cases predominantly occurring from July to November and peaking in September and October, as reported in hospital-based studies [7,8]. Other states such as Haryana, Chandigarh, Punjab, and Jammu and Kashmir have also documented scrub typhus cases [1]. The northern plains, though reporting fewer cases, have shown an increase in reported cases, indicating possible spread into previously less-affected geographic regions [9]. As scrub typhus is reported to public health authorities in India based on testing methods, data on scrub typhus-positive cases and deaths are available through surveillance systems. The Integrated Disease Surveillance Programme (IDSP) is an important programmatic source of scrub typhus data in India. According to data from the IDSP, India experienced a total of 127 reported outbreaks of scrub typhus from 2009 to 2023. Notably, there was one outbreak each in 2009 and 2010, while 2023 saw the highest number, with 27 outbreaks recorded, highlighting the need for strengthened surveillance and diagnostic infrastructure [10].

The disease manifests sporadically, often emerging in outbreaks during the monsoon and immediately afterward, which aligns with increased mite activity. In North India, scrub typhus accounts for 14% to more than 50% of cases of AUFI in tertiary-care AUFI cohorts during peak seasons [11,12]. These data highlight the crucial role of regional ecological factors in facilitating the life cycle of the vector and enabling disease transmission. Over the past 10 years, India has seen a rise in scrub typhus cases, which may reflect a combination of ecological shifts, growing urbanisation, and improved awareness [13-15]. The regional distribution of scrub typhus is presented in Table 1.

**Table 1:** Regional distribution of scrub typhus in North India. ELISA: Enzyme-linked immunosorbent assay; ICT: Immunochromatographic test; IFA: Indirect immunofluorescence assay; RDT: Rapid diagnostic test.

Author	Year	Patients with scrub typhus	Total patients in study	Diagnostic test used
Himachal Pradesh				
Vikrant S et al. [16]	2013	515 (37.7%)	1365	ELISA
Raina S et al. [17]	2018	262 (22.5%)	1164	ELISA
Uttarakhand				
Khan F et al. [18]	2015	692 (19.5%)	3540	ICT, ELISA
Ahmad S et al. [19]	2016	65 (27.8%)	233	ELISA
Bhargava A et al. [8]	2016	284 (22.2%)	1278	ELISA
Rawat V et al. [20]	2017	168 (59.1%)	281	IFA, ELISA, PCR
Punjab				
Oberoi A et al. [21]	2014	98 (12.6%)	772	ELISA
Haryana				
Jain D et al. [22]	2019	39 (16.9%)	230	ELISA
Uttar Pradesh				
Vivian Thangaraj JW et al. [23]	2017	40 (17.8%)	224	ELISA
Rizvi M et al. [24]	2018	91 (25.4%)	357	RDT, ELISA
Thangaraj JW et al. [25]	2018	155 (18.9%)	819	ELISA
Mittal V et al. [26]	2021	25 (20%)	125	ELISA
Husain U et al. [27]	2022	361 (20.7%)	1743	ELISA
Meena MK et al. [28]	2025	64 (15.6%)	410	ELISA
Chandigarh				
Kumar V et al. [29]	2014	49 (24.3%)	201	nPCR
Rauf A et al. [30]	2018	23 (10.5%)	217	ELISA

Risk factors for scrub typhus in Northern India

Numerous studies conducted in Northern India have identified various behavioural, environmental, and socioeconomic factors that contribute to the risk of scrub typhus infection. The main vectors are larval trombiculid mites, which thrive in scrub vegetation, grassy fields, and the litter of forest floors, enabling them to transmit the disease effectively to humans in these habitats [31-32]. A summary of the various risk factors reported from North India is shown in Table 2.

**Table 2:** Risk factors reported from North India.

Study / region	Study population / design	Key limitations	Risk factors (specific examples)	Message on risk profile
Jain D et al., (2019) [22]. Northern India (tertiary care centre)	Adults aged >14 years admitted with acute febrile illness; hospital-based observational series	Selection bias, hospital-based study	Rural backgrounds; working or moving through fields or scrub vegetation around villages	Affects rural adults exposed to vegetation and agricultural environments; village residents and field workers are at high risk
Bhargava A et al., (2016) [8]. Uttarakhand and adjoining Uttar Pradesh	Hospital-based cases from hilly Uttarakhand and western Uttar Pradesh plains	Retrospective study, hospital-based study, selection bias	Monsoon and post-monsoon peaks with dense vegetation and wet fields; farmers and outdoor labourers in fields and bushes	Concentrated in the monsoon and post-monsoon periods among outdoor and agricultural workers in hilly and peri-urban rural areas
Verma SK et al., (2021) [33]. Lucknow, Uttar Pradesh (tertiary care hospital)	Adult patients at a Lucknow tertiary hospital; observational series	Retrospective study, hospital-based study, selection bias	Rural residents from surrounding agricultural districts; exposure in fields and dense vegetation near homes	Clusters in rural catchment areas near cities with fields and scrub; needs community-level messaging
Bhat NK et al., (2014) [34]. North India (pediatric tertiary hospital)	Children with confirmed scrub typhus; observational cohort	Selection bias, hospital-based study	Rural and semi-rural areas; outdoor play or accompanying adults to fields or farms; vegetation and presence of animals around homes	Rural children spending time outdoors near fields, scrub, and animal sheds
Mukhopadhyay S et al., (2023) [35]. Western Uttar Pradesh and Rajasthan (Northwest India)	Pediatric patients; retrospective hospital-based review	Retrospective study, hospital-based study, selection bias	Peri-urban fields, vacant bushy plots, and animal shelters; extends to dry, arid, and urban-fringe areas	Re-emerging beyond rural forests to peri-urban and dry areas with chigger habitats

The key risk factors include the following: Occupational exposure, including farming, forestry, gardening, animal grazing, and fieldwork, increases contact with scrub vegetation that serves as a habitat for chiggers [23,36]. In rural areas, agricultural workers and forest dwellers account for a large proportion of cases. Environmental factors such as scrub vegetation in close proximity to residential areas, rodent infestations, and nearby dense undergrowth also significantly elevate the risk of infection [37]. Modifiable factors include behaviours that increase ground contact, such as sitting or lying on grass [12,38]. Although scrub typhus occurs across all age groups, adult males involved in outdoor occupations are more commonly affected. Nonetheless, several reports also highlight notable female involvement, primarily due to participation in agricultural activities [39,40].

These factors collectively sustain transmission in both endemic and emerging regions. Intensified outdoor agricultural activities, coupled with humid and cool environmental conditions, promote vector proliferation and likely increase exposure during peak monsoon periods.

Seasonal trends and climatic influences

Scrub typhus displays marked seasonality linked to the life cycle of vector mites and climatic conditions conducive to their breeding. Most studies document a steep increase in cases from July, with peaks occurring between September and November, and in some years extending into December, a pattern that is consistent across states [2,32,41]. This pattern aligns with the monsoon retreat and the subsequent cool, humid climate of the post-monsoon period. Environmental conditions, including temperature, rainfall, and humidity, are key climatic determinants affecting mite survival and disease transmission. Cooler temperatures between 15 and 28°C, combined with sufficient soil moisture and relative humidity, provide optimal conditions for mite proliferation. Some studies suggest that heavy rainfall may momentarily decrease vector contact, whereas moderate precipitation followed by sustained moist conditions favours the growth of chigger populations [37]. Seasonal patterns exhibit minor variations across different geographic areas in Northern India, yet consistently show the highest incidence of scrub typhus during the late monsoon and early winter months. Vector populations generally decrease with the arrival of severe cold winters or intensely hot and dry summers, leading to a natural reduction in case numbers during these times [42].

Clinical manifestations

Pathogenesis

The pathophysiology is dominated by diffuse necrotising vasculitis and perivasculitis of small blood vessels and capillaries in multiple organs. The organism infects endothelial cells and mononuclear phagocytes, triggering endothelial injury, increased vascular permeability, microthrombi formation, and a systemic inflammatory response that underlies many organ-specific manifestations such as pneumonitis, myocarditis, meningoencephalitis, and acute kidney injury (AKI) [43-45]. The strain heterogeneity (Karp, Kato, Gilliam, and numerous local genotypes) and host immune factors modulate disease severity and organ tropism, with Karp-like strains often associated with more severe systemic involvement. Moreover, Karp-like strain predominance has been observed in North Indian states [46,47]. Recurrent episodes and variable clinical presentations in endemic populations have also been reported [43,44].

*General and Systemic Clinical Features* 

The incubation period is usually 6-21 days, after which patients develop abrupt-onset high-grade fever with chills, headache, myalgia, and malaise, often indistinguishable from other acute febrile illnesses such as dengue, malaria, leptospirosis, or enteric fever [7,48]. Gastrointestinal symptoms, including anorexia, nausea, vomiting, abdominal pain, and sometimes diarrhoea, are common. In paediatric cohorts, vomiting and abdominal pain may be among the more frequent presenting complaints [34,49]. Physical findings frequently include tachycardia, relative bradycardia in some patients, hepatomegaly, splenomegaly, and tender lymphadenopathy, with hepatosplenomegaly reported in roughly one-third to nearly half of paediatric cases [34,43,49]. If untreated, fever typically persists for 2-3 weeks, and patients may progress in the second week to severe complications such as acute respiratory distress syndrome (ARDS), shock, meningoencephalitis, AKI, and myocarditis. Prompt doxycycline treatment often produces defervescence within 24-48 hours, which is an important therapeutic and diagnostic clue [43,48,50].

*Cutaneous and Mucosal Manifestations* 

The characteristic skin lesion is the eschar, a painless necrotic ulcer with a black crust and surrounding erythematous halo, resembling a cigarette burn, typically located in warm, moist, or concealed areas such as the axillae, groin, inframammary folds, waistline, or genital region [43,51]. The prevalence of eschar is highly variable, ranging from <20% to >70%, and may be lower in Indian populations, where dark skin, modest clothing habits, and lack of meticulous examination contribute to under-recognition. Nevertheless, diagnosis is strongly supported when an eschar is identified in a febrile patient from an endemic area [11,43,44]. A maculopapular or macular erythematous rash may appear on the trunk and spread centrifugally to the extremities, though it is inconsistently reported and may be transient or subtle [43,51]. Less common cutaneous manifestations include leukocytoclastic vasculitis, purpura, digital gangrene, and vasculitic phenomena such as Henoch-Schönlein purpura, reflecting the underlying small-vessel vasculitis [51,52]. Oral mucosal erythema or ulceration, conjunctival injection, and pharyngeal congestion are also described, but are non-specific and easily attributed to other febrile illnesses [43,48].

Ocular Manifestations

Ocular involvement is increasingly recognised and can occur during the systemic phase or, more commonly, in the convalescent period 2-4 weeks after the initial illness. Common findings include non-specific conjunctivitis, episcleritis, and anterior uveitis. Posterior segment manifestations such as retinitis, retinochoroiditis, retinal vasculitis, and optic disc oedema have been reported in selected tertiary cohorts. Rare but vision-threatening complications include optic neuritis, neuroretinitis, branch retinal artery occlusion, and macular oedema, sometimes occurring as part of a delayed immune-mediated inflammatory response rather than active infection. These complications may respond well to early systemic corticosteroids in addition to appropriate antibiotics. Because many ocular changes appear after systemic recovery, routine ophthalmologic screening in convalescent scrub typhus, particularly in severe or atypical cases, has been advocated to prevent permanent visual sequelae [53].

Respiratory and Pulmonary Involvement 

Respiratory manifestations span a spectrum from mild dry cough and dyspnoea to severe interstitial pneumonitis and ARDS. Pneumonia results from diffuse alveolar damage and pulmonary capillaritis, with chest imaging showing bilateral interstitial infiltrates or ground-glass opacities. In severe disease, frank ARDS with hypoxaemic respiratory failure may develop, often in the second week of illness [43,48,50]. ARDS due to scrub typhus has been well documented in case reports and ICU series, presenting with acute-onset tachypnoea, hypoxia, diffuse pulmonary opacities, and high ventilatory requirements, but it may improve rapidly with appropriate antibiotics and supportive care [48,50]. Respiratory involvement is an important independent predictor of ICU admission and mortality. Clinicians in endemic areas are encouraged to consider scrub typhus in any case of ARDS of unclear aetiology, particularly when accompanied by transaminitis, thrombocytopenia, or an eschar [43,45,50].

Cardiovascular and ECG Changes

Cardiovascular involvement is primarily a consequence of systemic vasculitis and capillary leak, leading to hypotension, shock, and occasionally myocarditis or pericardial effusion [43,45]. Clinical myocarditis may present with chest discomfort, elevated cardiac biomarkers, global left ventricular dysfunction on echocardiography, or arrhythmias [43,52]. In severe disease, distributive or mixed shock may occur due to vasoplegia, myocardial dysfunction, and hypovolaemia, often accompanying multiorgan failure [43-45].

Gastrointestinal and Hepatobiliary Manifestations 

Gastrointestinal involvement is frequent and may dominate the clinical picture, with nausea, vomiting, abdominal pain, diarrhoea, and hepatomegaly [34,43]. In many series from India and South-East Asia, hepatomegaly and splenomegaly are among the most consistent physical findings [34,43,49]. Laboratory evidence of hepatic involvement, including elevated transaminases, often with aspartate aminotransferase (AST) higher than alanine aminotransferase (ALT), hyperbilirubinaemia, and hypoalbuminaemia, is common [43-45]. Pancreatitis and gastrointestinal bleeding have been described as less common but serious complications [43,44,52].

Renal and Genitourinary Involvement 

Renal involvement ranges from mild reversible azotaemia to overt AKI requiring renal replacement therapy [43,44]. AKI results from a combination of prerenal factors, direct vasculitic injury to the renal microvasculature, and acute tubular necrosis [43-45]. In cohorts of severe scrub typhus admitted to the ICU, renal dysfunction is one of the most common organ failures, and higher creatinine levels have been associated with more persistent ECG abnormalities and worse outcomes [45]. Mild proteinuria and microscopic haematuria are frequently reported but are non-specific [43,44].

Neurological Manifestations 

Scrub typhus has emerged as a major cause of CNS infection in endemic regions, presenting with a spectrum of neurological manifestations ranging from mild headache to life-threatening meningoencephalitis [54,55]. Headache, altered sensorium, neck stiffness, and seizures are common in CNS scrub typhus; a meta-analysis of 1,221 patients with neurological involvement reported weighted pooled prevalence of fever as 100%, altered sensorium as 67%, headache as 65%, and neck stiffness as 57%, highlighting the resemblance to acute encephalitis syndrome [54]. Classical scrub typhus features such as eschar and lymphadenopathy are less frequently identified in CNS cases, with eschar present in only around 20% and lymphadenopathy in about 24%, which complicates clinical recognition [54,55]. Reported neurological complications include meningoencephalitis, acute disseminated encephalomyelitis, cranial neuropathies, cerebellitis, transverse myelitis, Guillain-Barré-like syndromes, and cerebrovascular events such as cerebral infarction, all attributable to a combination of vasculitis, immune-mediated injury, and endothelial dysfunction [45,52,54].

Haematological and Inflammatory Abnormalities

Haematological manifestations include anaemia, leukocytosis or leukopenia, and thrombocytopenia, the latter being particularly common and often severe in complicated cases [43,44]. In paediatric series from eastern and northern India, thrombocytopenia and features of capillary leak are frequent complications, correlating with disease severity and risk of ICU admission [34,49]. Coagulopathy and disseminated intravascular coagulation (DIC) may appear in severely ill patients, occasionally with clinical bleeding, and have been documented alongside shock in hospital-based cohorts [45,47]. Inflammatory markers such as CRP and procalcitonin (PCT) are typically elevated but non-specific, and mild hyponatraemia is also often observed, reflecting a systemic inflammatory and vasculitic process [43,44].

Severe Disease, Organ Dysfunction, and Predictors of Mortality 

Severe scrub typhus is characterised by one or more organ failures, including ARDS, shock, AKI, hepatic failure, meningoencephalitis, myocarditis, or DIC, frequently necessitating ICU admission [43,45]. A Taiwanese cohort study identified age ≥60 years, absence of eschar, and certain laboratory abnormalities as independent predictors of severe complications, suggesting that older patients without an evident eschar may be at particularly high risk of adverse outcomes in a non-Indian context [44]. In Indian ICU cohorts, respiratory failure, circulatory shock, renal dysfunction, CNS involvement, and the need for mechanical ventilation have been strongly associated with mortality, with reported ICU case fatality rates ranging from about 5% to over 20%, depending on the case mix and timing of therapy [43-45]. Meta-analyses focusing on CNS scrub typhus show case fatality ratios of around 3-6%, with higher mortality in paediatric cohorts, underlining the importance of early recognition and timely doxycycline or azithromycin therapy [49,54].

Clinical Manifestations in Children 

Children often present with acute febrile illness of 7-14 days’ duration, with prominent gastrointestinal symptoms such as vomiting and abdominal pain, along with cough, headache, and myalgia. Hepatomegaly, splenomegaly, and tender lymphadenopathy are common. Complications are frequent in hospitalised paediatric cohorts, with thrombocytopenia, meningoencephalitis, ARDS, bronchopneumonia, AKI, and myocarditis [34,49]. CNS involvement in particular appears relatively common in children with severe scrub typhus and should be suspected in any febrile child with encephalopathy and hepatosplenomegaly in endemic regions [34,49,54].

*Atypical and Rare Clinical Manifestations* 

Beyond the typical organ-system involvement, scrub typhus can present with a range of atypical manifestations, including isolated splenic infarction, myocardial infarction, pancreatitis, and cerebral infarction, all consistent with a vasculitic and prothrombotic pathogenesis [52]. Rare dermatological complications such as gangrene, vasculitic ulcers, and Henoch-Schönlein purpura have also been reported [51,52]. Atypical presentations without the classical triad of fever, rash, and eschar are common and often lead to diagnostic delay and inappropriate antibiotic use [11,47]. Early consideration of scrub typhus in any undifferentiated febrile illness with multisystem involvement in an endemic area remains critical to reduce morbidity and mortality [43,45,46].

Diagnostic overview and challenges

The diagnosis of scrub typhus relies on a combination of clinical assessment and laboratory confirmation tools. While the presence of an eschar is highly specific, it is variable in frequency (ranging from 7% to 97% depending on the region and strain) and is often missed in dark-skinned populations. Consequently, laboratory confirmation is essential. Current diagnostic strategies are broadly categorized into indirect serological methods, which detect host antibodies (IgM and IgG), and direct methods, which detect the pathogen or its genetic material. The choice of test often depends on the phase of illness, resource availability, and the specific strain distribution in the endemic area [56,57].

Indirect Methods of Diagnosis

Weil-Felix test: The Weil-Felix test relies on the cross-reactivity between *Orientia tsutsugamushi *antibodies and the *Proteus mirabilis* OX-K antigen. Historically, it was the only available test and remains in use in many resource-limited settings due to its low cost and ease of performance. However, its diagnostic utility is severely limited by poor sensitivity and specificity. It often yields false positives due to cross-reactivity with other Proteus infections and false negatives in the early stages of the disease. Despite these flaws, it is still utilized in rural primary care centres where advanced diagnostics are not available [58,59].

Immunofluorescence assay (IFA): The IFA is currently considered the "gold standard" for scrub typhus diagnosis. It detects IgM and IgG antibodies using antigen-coated slides, typically utilizing Karp, Kato, and Gilliam strains. A four-fold rise in antibody titres between acute and convalescent sera is considered confirmatory. However, single-titre cut-offs are often used in acute settings, though these cut-offs vary significantly by region, such as ≥1:64 in endemic areas versus ≥1:400 in non-endemic ones, to account for background immunity. The major limitations of IFA include the requirement for expensive fluorescence microscopes, the subjectivity of result interpretation leading to high inter-laboratory variability, and the need for paired sera to achieve high accuracy [56,60].

Indirect immunoperoxidase assay (IPA): The IPA is a modification of the IFA that replaces the fluorescent label with an enzyme peroxidase, allowing results to be visualized under a standard light microscope. This modification makes the assay more suitable for resource-limited settings lacking fluorescence equipment while maintaining sensitivity and specificity comparable to IFA. In comparative studies, IPA has served as a reliable confirmatory test, particularly when used alongside molecular methods. Like IFA, it allows for the quantification of antibody titres but shares similar limitations regarding the need for skilled personnel to interpret the staining patterns [61,62].

Enzyme linked immunosorbent assay (ELISA): ELISA has emerged as a preferred alternative to IFA for screening large numbers of samples. It utilizes recombinant antigens, often the 56-kDa type-specific antigen, to detect IgM, indicating acute infection, and IgG, indicating past or recurrent infection. Studies have demonstrated that ELISA offers high sensitivity (>90%) and specificity (>94%), as reported in several evaluations, often outperforming commercial rapid tests. It eliminates the subjectivity of microscopy-based methods and provides objective optical density readings. However, regional validation of OD cut-offs is necessary to distinguish active infection from background seropositivity in endemic populations [63,64].

Immunochromatographic test (ICT): ICTs, often in the form of lateral flow assays, function as rapid point-of-care (POC) diagnostics. These tests typically detect IgM, IgG, or total antibodies against recombinant *O. tsutsugamushi* antigens, including the 56-kDa protein, within 15-20 minutes. They are highly convenient for field use and rural hospitals, but their sensitivity can be lower than that of ELISA or IFA, particularly in the very early stages of infection. Nevertheless, their high specificity, ranging from 95% to 98%, makes them valuable tools for "ruling in" the diagnosis in acute febrile cases [65,66].

Direct Methods of Diagnosis

Bacterial isolation: Culturing *Orientia tsutsugamushi* is the definitive method for diagnosis but is rarely performed in routine clinical practice. The bacterium *O. tsutsugamushi* is an obligate intracellular pathogen requiring cell culture, typically L929 mouse fibroblast cell lines, or mouse inoculation. These techniques take approximately four weeks and require biosafety level 3 (BSL-3) containment facilities due to the risk of laboratory-acquired infection. Consequently, culture is restricted to research settings and reference laboratories for strain isolation and antibiotic susceptibility testing [67].

PCR: PCR offers high specificity (near 100%) by detecting *O. tsutsugamushi* DNA, commonly targeting the 56-kDa or 47-kDa genes in blood or eschar samples. It is most useful in the early phase of illness, during the window period before antibodies appear, when serological tests may be negative. Real-time PCR (qPCR) is more sensitive than conventional PCR and reduces the risk of contamination. However, sensitivity drops significantly, especially in blood, after the first week of fever as bacteraemia clears, limiting its utility in late-presenting patients. Therefore, the timing of sample collection is crucial depending on the type of test to be performed [68].

Future of scrub typhus diagnosis: The future of diagnosis is moving toward molecular POC testing and advanced genomic approaches. Loop-mediated isothermal amplification (LAMP) is a promising technology that eliminates the need for thermal cyclers. Recent studies indicate that LAMP achieves sensitivity comparable to PCR with the simplicity of visual readout, making it ideal for rural endemic areas. Additionally, metagenomic next-generation sequencing (mNGS) and clustered regularly interspaced short palindromic repeats (CRISPR)-based diagnostics are being explored to identify novel strains, addressing the issue of antigenic diversity [68,69].

Diagnostic challenges of scrub typhus

The primary diagnostic challenge is the immense antigenic heterogeneity of *Orientia tsutsugamushi*, which has many serotypes, including Karp, Gilliam, Kato, Kawasaki, and locally evolving strains. Serological tests based on prototype strains may fail to detect local variants, leading to false-negative results. Secondly, the scrub typhus infection criteria (STIC) remain debated; determining a universal cut-off titre for IFA is difficult in endemic areas. High background immunity in endemic areas often results in elevated baseline antibody titres in the general population, leading to difficulty in establishing a reliable cut-off titre. Finally, the window period creates a diagnostic blind spot in which serology is negative, but molecular tests are often unavailable in rural settings where the disease is most prevalent [70]. The comparative description of various diagnostic tests for scrub typhus is shown in Table 3, considering their specificity, sensitivity, turnaround time, key advantages, and limitations.

**Table 3:** Comparative description of various diagnostic methods for scrub typhus. *PCR sensitivity is high during the first week but declines as bacteraemia clears. TAT: Turnaround time; IFA: Immunofluorescence assay; ELISA: Enzyme-linked immunosorbent assay; ICT: Immunochromatographic test; POC: Point-of-care; LAMP: Loop-mediated isothermal amplification.

Method	Type	Sensitivity	Specificity	Typical lab TAT	Key advantages	Key limitations
IFA	Indirect	82-90%	High	2-4 hours	Gold standard; quantifies titre	Subjective; requires fluorescence microscope
Weil-Felix	Indirect	43-52%	Low	18-24 hours	Cheap; widely available	Poor accuracy; outdated
ELISA	Indirect	>90%	>94%	2-4 hours	Objective; high throughput	Batch processing needed; cut-off issues
ICT	Indirect	60-92%	95-98%	15-20 minutes	POC; simple	Lower sensitivity in early phase
PCR	Direct	Variable*	~100%	2-6 hours	Detects early infection in the window period	Expensive; lower sensitivity after the first week
LAMP	Direct	91-95%	High	<1 hour	Low-cost; molecular POC	Primer design complexity

Public health implications and challenges

Scrub typhus presents an escalating public health challenge in Northern India, driven by rising incidence, broad geographic distribution, diagnostic challenges, and the risk of severe outcomes. The disease disproportionately impacts rural and socioeconomically disadvantaged communities reliant on agriculture and forest resources.

Key implications include: (1) Many cases remain undiagnosed or are misdiagnosed because of nonspecific symptoms and limited laboratory facilities in rural areas, resulting in treatment delays, treatment failure, and increased morbidity and mortality [71]. (2) Seasonal peaks during the monsoon and post-monsoon periods can trigger outbreaks that place significant strain on regional health systems, highlighting the need for improved surveillance and rapid response mechanisms. (3) Increased hospitalisation, severe complications, and higher morbidity and mortality also create a substantial healthcare and economic burden on families and communities that depend on daily wages and farming income [72,73]. (4) In addition, the empirical use of antibiotics for febrile illnesses without a confirmed diagnosis may contribute to antimicrobial misuse and raise stewardship concerns if scrub typhus is not promptly recognised and treated with appropriate agents such as doxycycline or azithromycin [74]. However, the possibility of antimicrobial resistance remains theoretical, and resistance to doxycycline has not been reported. (5) Environmental and ecological factors, including deforestation, changes in land use, and climate fluctuations, further increase the complexity of vector ecology and reinforce the need for integrated, multidisciplinary public health strategies [41].

Effective interventions necessitate strengthening epidemiological surveillance systems, augmenting clinical management capacities, implementing comprehensive public health awareness campaigns, advancing targeted vector control strategies, and prioritizing research dedicated to vaccine development.

Control measures and recommendations

The control of scrub typhus relies on integrated strategies that simultaneously target the vector, reservoir hosts, and human exposure. These recommendations may include: (1) District-level access to ELISA and RDTs should be improved, along with clinician training in the AUFI algorithm, integration with IDSP reporting, and community education before the monsoon season. (2) Personal protective measures, such as the use of insect repellents, wearing long-sleeved clothing, and avoiding sitting or lying directly on the ground, particularly in scrub-inhabited areas during peak seasons, are also important [42]. (3) Environmental management strategies, including clearing vegetation around homes, controlling rodent populations, and maintaining clean surroundings, can help reduce breeding sites for disease vectors [75]. (4) Community health education should focus on raising awareness about risk factors, early signs of disease, and the importance of seeking prompt medical attention. (5) In addition, strengthening laboratory diagnostic capacity at district and sub-district levels, together with systematic case reporting, is essential to improve outbreak detection and optimize resource allocation. (6) Clinical training programmes for healthcare providers should focus on recognising scrub typhus, guiding early testing, and ensuring the prompt administration of appropriate antimicrobial therapy. (7) Support should also be given to research on local vector ecology, pathogen strain diversity, and vaccine development, including efforts to conduct vaccine trials for the sustainable reduction of disease incidence [76]. Effective control of scrub typhus requires coordinated efforts across the public health, veterinary, environmental, and agricultural sectors through multi-sectoral collaboration.

Limitations of the study

There were certain limitations associated with this review. As this was a narrative review, it may be prone to selection and confirmation bias. In addition, because this was a regional study, the findings cannot be generalised to the entire population.

## Conclusions

Scrub typhus is an emerging and serious public health issue in Northern India, characterized by unique regional, ecological, and seasonal variations. The disease predominantly affects rural communities exposed to scrub vegetation during and after the monsoon season. Despite its significant morbidity and outbreak potential, scrub typhus is often under-recognized due to diagnostic difficulties and symptom overlap with other febrile illnesses. Tackling this disease requires strengthening diagnostic and surveillance systems, raising awareness among the public and healthcare providers, promoting preventive practices, and enhancing integrated vector and environmental control strategies. Continued research is vital to deepen understanding of the disease epidemiology and to support the development of effective vaccines, along with district-level diagnostic strengthening and standardized IFA cut-offs to provide clearer, actionable guidance. Coordinated and sustained efforts can markedly reduce the burden of scrub typhus and improve health outcomes in affected areas.
